# Mapping inertial migration in the cross section of a microfluidic channel with high-speed imaging

**DOI:** 10.1038/s41378-020-00217-y

**Published:** 2020-11-16

**Authors:** Jian Zhou, Zhangli Peng, Ian Papautsky

**Affiliations:** Department of Bioengineering, University of Illinois at Chicago, Chicago, IL USA

**Keywords:** Engineering, Physics

## Abstract

The wide adoption of inertial microfluidics in biomedical research and clinical settings, such as rare cell isolation, has prompted the inquiry of its underlying mechanism. Although tremendous improvement has been made, the mechanism of inertial migration remains to be further elucidated. Contradicting observations are not fully reconciled by the existing theory, and details of the inertial migration within channel cross sections are missing in the literature. In this work, for the first time, we mapped the inertial migration pathways within channel cross section using high-speed imaging at the single-particle level. This is in contrast to the conventional method of particle streak velocimetry (PSV), which provides collective information. We also applied smoothed particle hydrodynamics (SPH) to simulate the transient motion of particles in 3D and obtained cross-sectional migration trajectories that are in agreement with the high-speed imaging results. We found two opposing pathways that explain the contradicting observations in rectangular microchannels, and the force analysis of these pathways revealed two metastable positions near the short walls that can transition into stable positions depending on the flow condition and particle size. These new findings significantly improve our understanding of the inertial migration physics, and enhance our ability to precisely control particle and cell behaviors within microchannels for a broad range of applications.

## Microfluidics: Mapping the migration of individual particles within microchannels

For the first time, the pathways of inertial migration in the microchannel cross section have been mapped with high-speed cameras at the single-particle level. Inertial migration offers a way to manipulate neutrally buoyant microparticles or cells towards the walls of microchannels, and through orthogonally placed channels. Despite this phenomenon being increasingly exploited in biomedical research for applications such as cell sorting, the fundamentals of how fluid forces in inertial microfluidics interact to facilitate the lateral movement has only now been elucidated. Jian Zhou, Zhangli Peng, and Ian Papautsky, of the University of Illinois at Chicago, United States, used high-speed photography to map the trajectories of single particles down 10mm-long microchannels. They corroborated their observations with 3D fluid dynamics simulations. In doing so, they resolved previously-conflicting observations, and discovered newly-observed and unexpected particle migration behaviors.

## Introduction

The rapid development of inertial microfluidics in the past decade has attracted significant interest from a variety of fields and has triggered an avalanche of applications in biomedical research and clinical diagnostics^1–3^. Nearly a decade ago, our group^4,5^ and Di Carlo et al.^6,7^ showed the effective manipulation of microparticles flowing in simple microchannels for focusing and filtration without sophisticated external control. Since then, increasing attention has been poured into the field, and numerous investigators have focused on unleashing the potential of inertial microfluidics. Numerous microfluidic systems based on inertial migration have been reported to tackle a wide range of challenges, including label-free cell sorting^8–10^, efficient focusing for flow cytometry^11–13^, cell mechanotyping^14,15^, and the high-throughput isolation of circulating tumor cells (CTCs) for liquid biopsy applications^16–22^.

While new applications of inertial microfluidics continue to emerge, the mechanism underlying inertial migration remains unclear. Inertial migration refers to the phenomenon where neutrally buoyant particles or cells suspended in channel flow migrate laterally and across streamlines, orthogonal to the flow direction, at moderate Reynolds numbers (*Re*) where fluid inertia dominates the particle motion. Inertial migration was first reported by Segré and Silberberg^23^^,^^24^ over half a century ago when they observed the lateral migration of particles in a small pipe, resulting in the formation of a particle-concentrated annulus near the wall. It is now well accepted that particles in pipe flow are mainly subjected to two types of forces: viscous drag (*F*_D_) and inertial lift (*F*_L_). While the former keeps particles within streamlines and drives particle motion streamwise, the latter is responsible for their cross-streamline migration. The formation of the particle annulus is attributed to a pair of inertial lift forces^1,25^—the shear-induced lift force (*F*_s_)^25,26^ that drives particles away from the channel centerline and the wall-induced lift force (*F*_w_)^27,28^ that acts on particles in the opposite direction as particles approach the capillary wall. The balance of these two inertial forces results in stable equilibrium positions at approximately 0.2*D* away from capillary wall (where *D* is the capillary diameter). However, this force balance is insufficient to explain the reduced equilibrium positions observed in radially asymmetric channel cross sections, such as square^25,28^, rectangular^12,29^, and triangular^30–32^ microchannels.

In 2013, we proposed a two-stage model for the inertial migration of particles^33^, which explained the occurrence of the reduced equilibrium positions. We introduced an additional inertial force, termed the rotation-induced lift force (*F*_Ω_), which is unlike *F*_s_ and *F*_w_, that acts parallel to the channel sidewall when particles are in close proximity to the wall. Thus, *F*_s_ is responsible for particle migration toward channel walls (Stage I), and *F*_Ω_ further drives the particles to their equilibrium positions centered on each face of the channel cross section (Stage II). While our model reconciles the discrepancy between the early observations, there are other migration behaviors that it cannot explain. For example, the presence of two-side streams of particles has been observed before full focusing is reached in straight microchannels^12,34^. These side streams compromise the focusing quality and separation efficiency in particle and cell cytometry applications. Elucidating the mechanism behind such an observation will reveal the conditions that either stabilize or eliminate the side streams for improved performance in the spatial manipulation of particles and cells. Furthermore, the model was based on the collective migration of particles observed via particle streak velocimetry (PSV), which can conceal details of particle migration, especially of some rare events. Thus, to resolve the discrepancies and to develop a more complete understanding of inertial migration, the dynamics in a channel cross section must be resolved. Koh et al.^35^ used a microchannel with an embedded microprism and simultaneously observed particles in top- and side-view images, which enabled the determination of particle positions in a channel cross section. Later, Hood et al.^36^ derived particle positions in a channel cross section using the velocity-based reconstruction method and measured the migration velocity. However, neither of these studies resolves the migration dynamics in the cross section, which remains a challenge and has not yet been achieved.

In this work, we map the dynamics of inertial migration within the channel cross section using high-speed imaging at the single-particle level and, for the first time, report on observations of unexpected new migration behaviors. Tracking migration trajectories of individual particles, we explicitly show their lateral displacement, providing the missing evidence in inertial microfluidics. Coupling the migration trajectories in the top- and side-view imaging of our microchannel, we derived particle migration pathways within the channel cross section for the first time. Our mapped paths suggest heterogeneous migration, which is dependent on the particle location within the cross section. These results are consistent with the numerical results obtained using a smoothed particle hydrodynamics (SPH) code, which predicts the transient pathways of particles instead of only predicting the force distribution in channel cross sections^37^. Analyses of the paths and the underlying forces revealed two metastable focusing positions near the sidewalls. This result explains the earlier observations of side streams and the evolution of focusing positions in rectangular channels. These new findings suggest a temporal dependence of the side streams. Overall, this work improves our understanding of the inertial migration mechanism and enhances our ability to precisely manipulate particles and cells within microchannels to meet the needs of a wide range of biomedical applications.

## Methods

### Experimental

#### Microfabrication

Microchannels were fabricated in polydimethylsiloxane (PDMS) using soft photolithography with dry film materials^38^. Briefly, a dry film (ADEX 25 & ADEX 50, DJ MicroLaminates Inc., MA, USA) was used to pattern microchannels on a 3″ silicon wafer by conventional photolithography. PDMS (Sylgard 184, Dow Corning®, MI, USA) was cast on the wafer and peeled after a 2 h cure on an 85 °C hotplate. Replicated straight channels (50 μm × 25 μm and 25 μm × 50 μm in width × height format) in PDMS were bonded to 1″ × 3″ glass slides (Fisher Scientific, MA, USA) using surface plasma treatment (PE-50, Plasma Etch Inc., NV, USA.). The inlet and outlet ports were punched manually using stainless flat head needles. The channels were 10 mm in length. The sample solution was injected into the PDMS device with a syringe pump (Legato 200, KD Scientific Inc, MA, USA) to sustain a stable flow rate. The loaded syringe was connected to 1/16″ Tygon® tubing (Cole-Palmar, IL, USA) using proper fittings (IDEX Health & Science LLC, WA, USA) and then secured to the device inlets.

#### Sample preparation

Particles with diameters of 18.7 μm (Polysciences Inc.) were diluted in deionized (DI) water to a volume fraction of 0.025% to avoid particle–particle interactions. A small drop of TW-80 was added to prevent particle aggregation and sticking to the channel walls. The flow rates were fixed at 112.5 μL/min, corresponding to *Re* = 50. The Reynolds number is given as *Re* = *ρU*_f_*D*_h_/*µ*, where *ρ* and *µ* are the fluid density and viscosity, respectively, *U*_f_ is the average velocity of flow, and *D*_h_ is the hydraulic diameter of the microchannel (*D*_h_ = *2wh/*(*w* + *h*)). Low aspect ratio (50 μm × 25 μm) and high aspect ratio (25 μm ×50 μm) channels were used to investigate the 3D dynamics of particle migration in the first 1-mm downstream length. The aspect ratio was defined as the ratio of the channel height (*h*) to the width (*w*), AR = *h*/*w*. At *Re* = 50, the entrance length (*L*_e_) required for the full development of the flow was ~108 μm (*L*_e_ = 0.065*Re* × *D*_h_)^39^.

#### High-speed imaging and data analysis

The particle flow was imaged at the beginning of the microchannel placed on the stage of an inverted microscope (IX-83, Olympus Inc.). Images were acquired using a high-speed camera (Mini AX200, Photron USA, Inc.) with observation windows set to 1024 μm × 128 μm (×20 objective). The frame rate was 25,000 or 30,000 fps, and the exposure time was 1 μs. The trajectories of individual particles were obtained by stacking consecutive frames using ImageJ^®^ software. The downstream and lateral positions of particles at each frame were measured manually using PFV software (Photron USA, Inc.). The data points of these measurements within the first 108 μm downstream length were omitted to avoid the impact of the entrance length^39^ on the migration when migration paths were inferred.

### Computational

We applied the smoothed particle hydrodynamics approach to simulate the transient motion of a rigid sphere in a 3D rectangular channel under certain pressure gradients. Compared to traditional mesh-based methods, such as finite element and finite volume methods, it is relatively easier to implement the modeling of fluid-particle interactions in SPH. Furthermore, SPH has been successfully applied to study particle migrations in Newtonian and viscoelastic flows, and its validations and advantages have been clearly demonstrated in many cases. Fluid–solid interactions were solved by enforcing the non-penetration and non-slip boundary conditions on a rigid sphere surface in SPH. SPH is a classical Lagrangian particle simulation technique for fluid dynamics that solves the Navier–Stokes equations by discretizing the fluid domain using SPH particles. The SPH particles move with the velocity of the flow fields. The momentum is conserved using pairwise interactions between the SPH particles.

#### SPH model

In the current study, we used a version of weakly compressible SPH with a formulation similar to the original work by Gingold and Monaghan^40^ and Lucy^41^. The compressibility of the flow was within 3% by setting the sound speed more than 20 times higher than the maximum velocity in the simulation. Viscous forces were added using Morris’s formula^42^, and the water viscosity at room temperature was applied. We used a Lucy smoothing kernel^41^ with a smoothing length large enough to include sufficient neighboring particles. A velocity Verlet algorithm was applied to update the position and velocity of each particle. The density of fluid SPH particles was set to the water density (1000 kg/m^3^), and the density of the SPH particles in the rigid sphere was set to the sphere density in the experiment (1050 kg/m^3^).

#### Boundary conditions

To ensure the non-penetration and non-slip boundary conditions on the wall and rigid sphere surface, we used an approach of extrapolating the velocity to the wall particles based on the distances of the wall and fluid particles to the boundary^42^. Body forces were added to generate the pressure gradient and flow rate was measured in the experiments. The rigid spheres consist of the spherical regions of SPH particles, and the positions, velocities, and angular velocities of the rigid spheres were updated based on rigid body dynamics. All these approaches were implemented in LAMMPS.

#### Simulation setup

We used a periodic boundary condition in the flow direction (*X* direction) and fixed wall boundary conditions in the *Z* and *Y* directions. We built a regular orthogonal uniform lattice of SPH particles in an initial configuration and assigned a layer of wall particles with enough depth to be fixed. Then, we assigned a spherical region of SPH particles as a rigid group to represent the rigid sphere and applied the rigid body dynamics integrator in LAMMPS to update its motion. The center of mass and other properties, such as the linear and angular velocities and the force and moment on the rigid sphere, were recorded during the simulation.

## Results

### Location-dependent migration

**Fig. 1:**
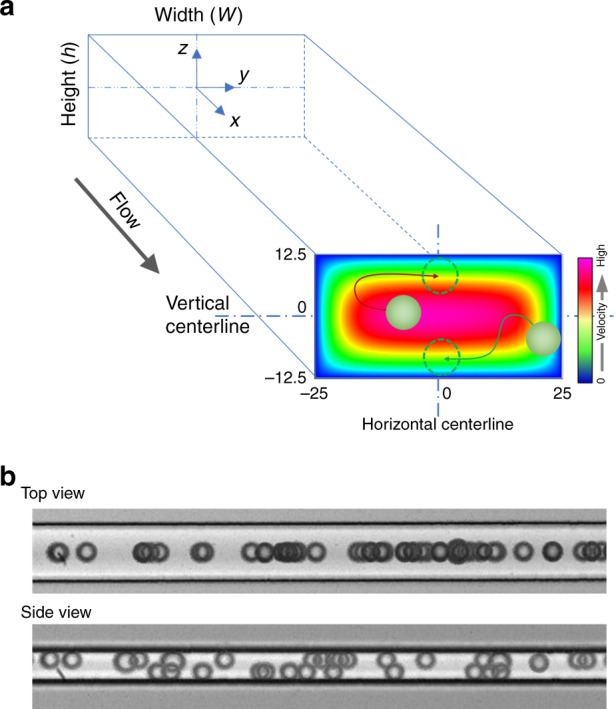
Channel diagram and particle migration dynamics. **a** Graphical illustration of the particle migration within the cross section. Red and green curves illustrate two of the unexpected migration paths of particles in the cross section. Dashed circles indicate the final equilibrium positions. The heat map at the cross section indicates the velocity distribution. **b** Top- and side-view images of particles fully focused 3 mm downstream

Considering the finite field of view (FOV) of our microscope and high-speed camera, the dimensions of the microchannel and particle size must be carefully selected to observe the lateral displacement of single particles at sufficiently high resolution and without microscope stage movement. A microchannel with a cross section of 50 μm × 25 μm (Fig. 1a) was selected to examine the inertial migration dynamics of 18.7-μm-diameter particles. This combination of channel and particle sizes permitted rapid lateral migration and full focusing of particles in a short, 2.8 mm downstream length at *Re* = 50. This length was calculated based on the two-stage model^29^ and confirmed using stacked images of the first 4 mm of the channel length (Fig. S1). A pair of channels with reciprocal dimensions (50 μm × 25 μm and 25 μm × 50 μm) were fabricated to permit the top- and side-view imaging of the particle migration. Representative images in Fig. 1b illustrate the focusing of particles into two distinct streams 3 mm downstream. Next, we used this approach to investigate the cross-sectional migration dynamics at a single-particle level.

**Fig. 2:**
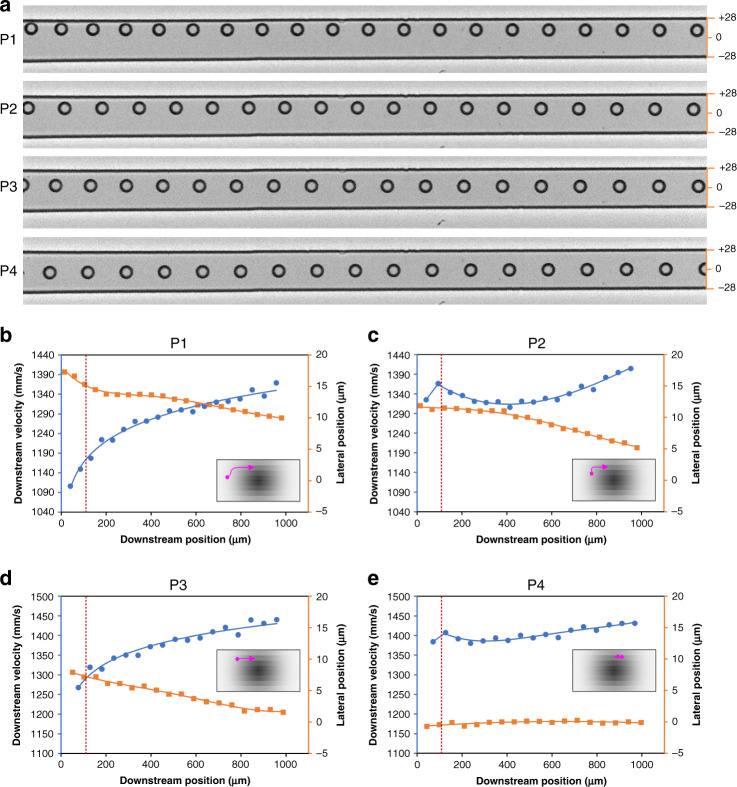
High-speed imaging reveals the migration behavior of individual particles observed in the channel top view. **a** Stacked bright field images capturing migration trajectories of four representative particles (P1, P2, P3, and P4). The channel width expanded to 56 μm due to fluid pressure in these experiments. A frame rate of 25,000 and exposure of 1 μs were used to acquire images. **b**–**e** Measurements of the downstream velocity and lateral position as a function of the downstream position for the four particles in panel **a**. Insets graphically illustrate the inferred migration paths (pink) within the channel cross section, where the grayscale indicates the approximate downstream fluid velocity magnitude. The dotted red lines indicate the channel entrance length required for full flow development

Our results offer direct evidence of the lateral inertial migration of particles in a microchannel. As shown in Fig. 2a, particles near the channel sidewalls show phenomenal lateral displacements toward the width center of the channel (top view), which corresponds to Stage-II migration and correlates with our previous PSV results^33^. The migration of individual particles was quantified in terms of the downstream position, downstream velocity, and lateral position. We analyzed two sets of four particles with different initial positions from the top- and side-view imaging. We begin our discussion from top-view imaging, as it exhibits a larger displacement and is easier to observe.

We found that inertial migration is heterogeneous, depending on the particle’s initial position within the channel cross section. For a particle near the sidewall (Fig. 2a), its downstream velocity increased as it traveled downstream; this was anticipated, as the particle crossed streamlines toward the high velocity region near the channel center due to the parabolic velocity profile in the channel flow. Plotting the downstream particle velocity as a function of the downstream position yields a logarithmic curve (Fig. 2b), with a rapid initial increase followed by saturation. The lateral migration of the particles toward the channel centerline also exhibited a multistep shape. Overall, the particle migrated from 17 to 10 μm, yielding an average migration velocity of 9.7 mm/s. However, at 14 μm, the particle maintained its lateral position for a short time before resuming a steady migration toward the horizontal centerline. This non-steady lateral displacement, combined with a logarithmic increase in the downstream velocity, suggests that the migration within the cross section is not unidirectional. One can infer that the particle initially migrated toward the channel horizontal centerline while accelerating downstream, followed by vertical migration, and finally resuming migration along the horizontal wall toward the centerline. This inferred migration path is graphically illustrated in the inset of Fig. 2b.

Coupling the variation in the downstream velocity and the change in the lateral position, we can also infer the migration paths of the other observed particles. Particle P2, initially between the channel wall and centerline, slowed slightly before steadily accelerating. Meanwhile, its lateral position remained unchanged even during the slowdown, which suggests that the particle was migrating vertically toward the low velocity region near the sidewall. Hence, the two-step migration path is as follows: first up the channel sidewall and then toward the channel centerline (Fig. 2c, inset). Note that we omit data points within the entrance length of the channel (before the dotted red line in Fig. 2b–d) when inferring migration paths to avoid the effect of the flow development. While particle P3 exhibited similar logarithmic behavior to P1 in terms of the downstream velocity, its lateral migration appears to be steady, which suggests that the particle was already near the wall and thus mainly migrated toward the channel centerline (Fig. 2d, inset). Particle P4, which was already near the horizontal centerline on entering the channel, exhibited little lateral migration (<1 μm toward width center) and only a slight change in the downstream velocity. This slight change suggests that the particle was already near its equilibrium position (Fig. 2e, inset).

**Fig. 3:**
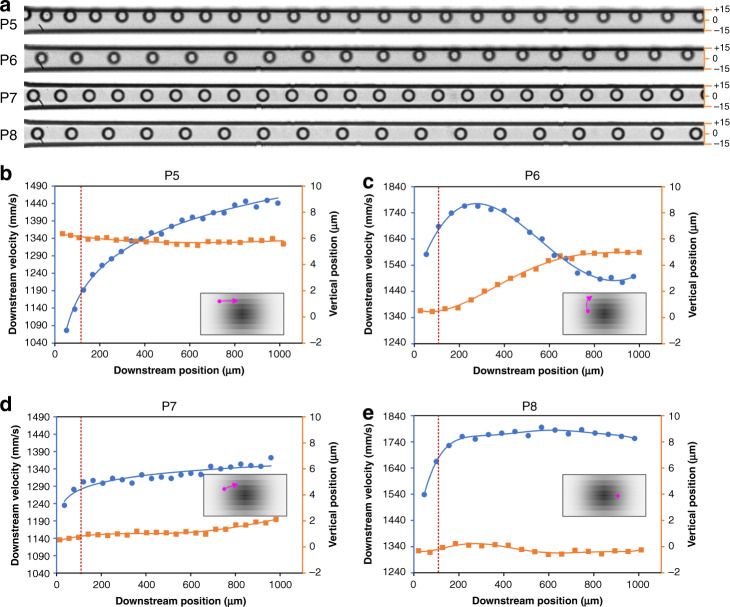
High-speed imaging reveals the migration behavior of individual particles observed in the channel side view. **a** Stacked bright field images capturing migration trajectories of four representative particles (P5, P6, P7, and P8). The channel height expanded to 30 μm due to the fluid pressure in these experiments. A frame rate of 30,000 and exposure of 1 μs were used to acquire images. **b**–**e** Measurements of the downstream velocity and vertical position as a function of the downstream position for the four particles in panel **a**. Insets graphically illustrate the inferred migration paths (pink) within the channel cross section, where the grayscale indicates the approximate downstream fluid velocity magnitude. The dotted red lines indicate the channel entrance length required for full flow development

Similarly, particle migration in the cross section can also be inferred from the high-speed imaging in the side view. Particle trajectories from side-view images in Fig. 3a reveal the off-center migration toward channel walls in general, but not without heterogeneity depending on the cross-sectional location. Particle P5 initially near the long channel wall exhibited a small displacement (<1 μm), while its downstream velocity increased by ~34%, with the velocity curve resembling the parabolic profile of channel flow. This behavior suggests that the cross-streamline migration of the particle was mainly along the long horizontal channel wall (Fig. 3b, inset), similar to that of the particle in Fig. 2d. Additionally, this behavior is clear evidence of Stage-II migration^33^ and is further corroborated by the particle becoming out of focus as it moves downstream (Fig. 3a), suggesting vertical displacement. Particle P6, initially between the wall and channel center, migrated toward the channel wall undergoing Stage-I migration (Fig. 3c), as indicated by the significant decrease in the downstream velocity, since the velocity near the wall is much lower. The initial increase in the velocity is due to the effect of the channel entrance length. Mixed migration behavior was also observed, with particles undergoing both Stage-I and Stage-II migration at the same time. For example, particle P7 was the case between P5 and P6. While P7 migrated toward the wall similar to P7, its downstream velocity increased slightly, which is similar to P5 (Fig. 3d, inset).

Surprisingly, particle P8 exhibited very little change in both the lateral position and downstream velocity beyond the channel entrance length (Fig. 3e, inset). The lateral displacement of P8 was less than 1 μm. Its high downstream velocity (~1780 mm/s) suggests that the particle was farther away from the sidewall. Noticing that this behavior of P8 seems to contradict our two-stage migration model has led us to examine individual particle trajectories, which has uncovered more distinct migration behaviors, as discussed below.

### Unexpected particle behavior

**Fig. 4:**
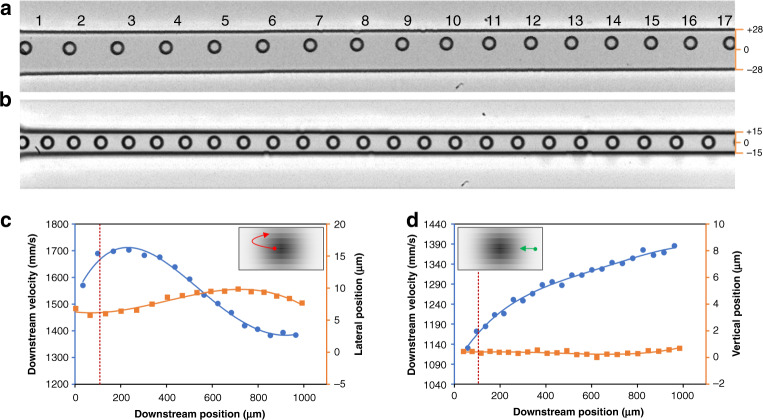
Unexpected migration behavior. **a** A particle trajectory illustrating an outward migration prior to a subsequent inward migration toward the channel center in the top-view high-speed images. **b** A particle initially located at the channel vertical centerline exhibits a slight vertical migration throughout the observation window. **c**, **d** Corresponding measurements of the downstream velocity and lateral position as a function of the downstream position. Insets graphically illustrate the inferred migration paths (red and green) within the channel cross section, where grayscale indicates the approximate downstream fluid velocity magnitude. The dotted red lines indicate the channel entrance length required for full flow development

Unexpected migration behavior was observed from multiple particles, with representative results shown in Fig. 4. For example, while most particles exhibited migration toward the channel centerline in top-view imaging, as discussed above, some particles displayed an unexpected migration path. As shown in Fig. 4a, a particle initially near the channel centerline first migrated toward the channel sidewall before reversing direction of its migration and beginning to return toward the centerline. At downstream position #11 in Fig. 4a, the particle reached its farthest lateral position, which was ~4 µm closer to the wall than its initial position. Afterward, there was little lateral displacement for the following four downstream positions (#11–14), as shown in Fig. 4c. Meanwhile, its downstream velocity decreased concurrently, suggesting that the particle was mainly experiencing vertical migration during this period. The velocity remained largely unchanged even when the particle progressed toward the horizontal centerline, suggesting that the particle migrated back through different vertical planes (Fig. 4c, inset). Such migration behavior is different from the vast majority of the particles, as discussed above and as shown in Fig. 2. Such behavior has not been reported in the literature before and is unexpected based on the well-accepted explanation of migration dynamics^1,33^.

Another type of behavior exception was observed in the side-view imaging where a particle was initially located in the channel center plane (Fig. 4b). While its trajectory is quite similar to that of particle P8 in Fig. 3e, the two particles had sheer differences in the downstream velocity. Unlike particle P8, the downstream velocity of this particle increased steadily, yielding a logarithmic increase in the velocity (Fig. 4d) that resembles the classic parabolic velocity profile of channel flow. This distinctive downstream velocity curve suggests that the particle migrated up the velocity gradient away from the nearest short wall (Fig. 4d, inset). This observation is again unexpected based on the existing theory of inertial migration.

### General migration pattern

**Fig. 5:**
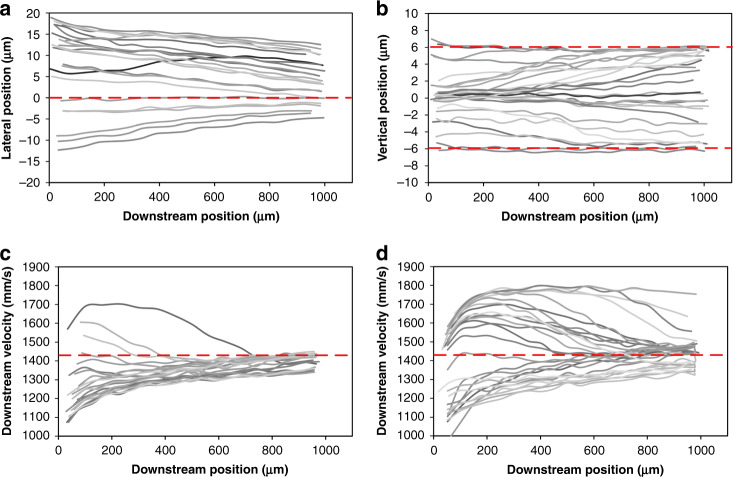
Migration trajectories and downstream velocities of the particles. Particles aggregate laterally toward the channel horizontal centerline (**a**) and vertically toward the long channel walls (**b**). The dashed red lines indicate the focusing positions. The imaging channel in the top view (**c**) and side view (**d**) shows the convergence of the downstream velocity. The downstream velocities and trajectories of *n* = 23 particles were considered in the top view, and *n* = 27 particles were considered in the side view

Despite some unexpected migration behavior, our two-stage model generally holds up. This model is illustrated by the aggregate trajectories and measurements of downstream velocities of 29 particles in Fig. 5. In the top-view images, particles migrated toward the horizontal centerline, and all trajectories converged eventually (dashed line in Fig. 5a), which is consistent with Stage-II migration in our earlier model. Since rotation-induced lift force (*F*_Ω_) dominates particle migration in this stage, this is a convergent force that brings particles toward the channel centerlines. In the side-view images, particles migrated away from the vertical centerline, and trajectories merged into two lines near the top and bottom walls (dashed lines in Fig. 5b). This divergent trend of trajectories from the channel centerline is in agreement with the Stage-I migration of our early model. As shear-induced lift force *F*_s_ dominates such a migration, this is a divergent force that drives particles away from the channel centerlines. Upon completing both stages, all particles eventually focused in two positions in the horizontal centerline near the top and bottom walls, where their downstream velocity converged to ~1440 mm/s in both the top- and side-view images (Fig. 5c, d).

The trajectories and downstream velocity curves in Fig. 5 suggest that particles were not uniformly distributed in the channel cross section at the channel entrance. During the experiments, we observed very few particles initially located within the vertical range of 2–5 μm off the centerline (Fig. 5b). We also only recorded a few particles with downstream velocities above the dashed red line in Fig. 5c. These observations suggest the nonuniform distribution of particles in the cross section initially, likely due to a short contraction channel segment prior to the main channel entrance. The contraction channel segment was necessary to gently guide the particles into the main channel, although some particles began prefocusing in this channel segment as early as 450 μm ahead of the main channel^36^. Additionally, considering their large diameter, the particles may preferentially migrate in the cross section during flow development within the entrance length.

### Cross-sectional migration paths

**Fig. 6:**
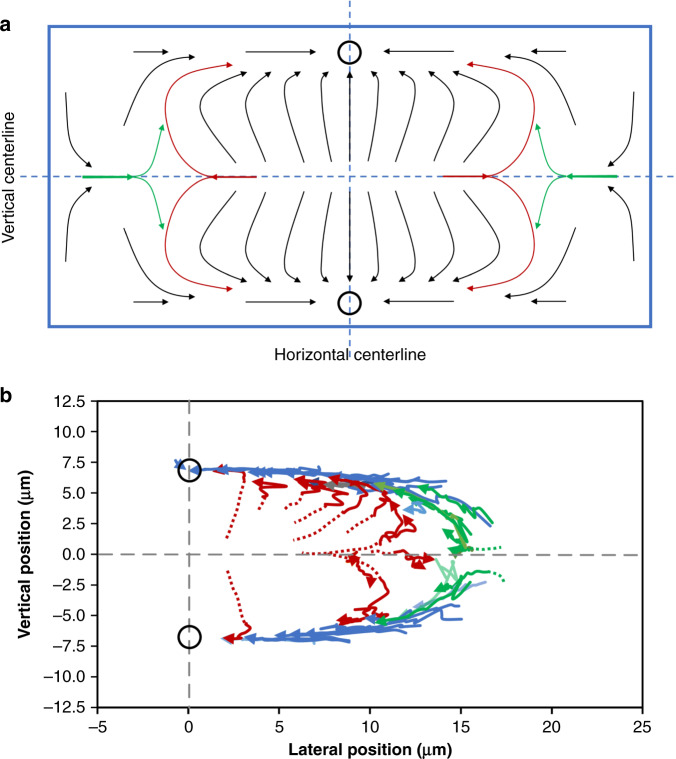
Inertial migration of particles in the rectangular cross section of a microchannel. **a** Graphic illustration of the inferred migration paths in the channel cross section. The open circles indicate the final focusing positions. The schematic not drawn to scale. **b** Migration trajectories of particles (*n* = 50) in the right half of the channel, derived from top- and side-view imaging. The dotted lines are added for visualization

Coupling the observations in the top and side views, we can resolve particle migration paths within the channel cross section (Fig. 6). The revelation of particle migration behavior orthogonal to the flow direction is essential for deciphering the full dynamics of inertial focusing and thus in reconciling contradicting observations. Since the direct observation of particle movement in the channel cross section is highly challenging, as particles only appear once in a focal plane, our observation through top-view and side-view imaging permits us to reconstruct the migration pathways of particles in the channel cross section (Fig. 6a).

The migration paths of particles located differently in cross section can be distinct despite particles largely following the two-stage migration model. In general, most particles migrate toward the long walls (widths) before changing their migration directions toward the horizontal centerline. High-speed imaging at the single-particle level reveals the details of particle behavior during Stage-I migration, including the migration along the red paths. These details suggest that the migration direction is not uniform in Stage I and that the direction can be the combinatory result of the vertical and horizontal migrations. For most particles, vertical migration toward the long walls dominates their overall migration behaviors as they very rapidly complete the Stage-I migration (Fig. 4d and Fig. S1). However, horizontal migration toward the sidewalls becomes significant for the particles near the vertical centerline (red paths). The particles near the long walls are mainly subjected to horizontal migration toward the horizontal centerline (Stage-II migration). While some particles near the sidewalls move toward the vertical centerline, they gain momentum toward the center axis when they reach the vertical centerline (green paths), in contrast to the red paths. The opposing migration paths (green and red) in the cross section suggest temporary (metastable) focusing positions near the sidewalls, which will be discussed in detail below.

As we do not have direct access to the particle cross-sectional position, we use a semiquantitative method to resolve the migration paths of the particles based on the lateral/vertical position and the corresponding downstream velocity. We assume that the particles follow the parabolic velocity profile of the channel flow, and by matching the particle downstream velocity with the fluid velocity, the paths in the cross section can be elucidated. With the maximum fluid velocity (2050 mm/s) measured using 2-μm-diameter particles (Fig. S3), the fluid velocity in the cross section can be fully resolved. Thus, the vertical position of a particle in the top-view image can be determined using its lateral position and downstream velocity. Similarly, the lateral position of a particle in the side-view image can be obtained using its vertical position and downstream velocity. Figure 6b shows the calculated migration paths of 50 particles, which match the cross-sectional migration pathways in Fig. 6a.

However, there are limitations to our analysis. First, we assume that the particle downstream velocity matches that of the carrier fluid. This assumption is generally valid if the particle size is significantly smaller than the channel dimensions, i.e., *a*/*D*_h_ « 1, such as the case in particle image velocimetry. Second, previous work suggests that particles in the inertial flow lag the flow^43^. In our case (*a*/*D*_h_ = 0.56), we found that the particle velocity resembles the general parabolic flow profile (Fig. S3) and that particles appear to lag the fluid. The lag becomes significant at a high downstream velocity (>1600 mm/s). This lag of particles behind the flow can result in an inaccuracy in the cross-sectional locations of the derived paths. Thus, the data with a downstream velocity >1600 mm/s were not used in deriving the paths in Fig. 6b. Additionally, channel deformation was ignored in the derivation. For these reasons, we consider our derived paths in Fig. 6b semiquantitative. In future work, the approach using a microprism similar to Koh et al.^35^ can be adapted for the simultaneous observation of the lateral and vertical positions of single particles in an inertial flow. In this way, the migration pathways in the cross section can be analyzed quantitatively to provide further insight. In the next section, we discuss the numerical simulation that we used to further confirm the inferred migration pathways in the cross section.

### Numerical simulations of migrations in the cross section

**Fig. 7:**
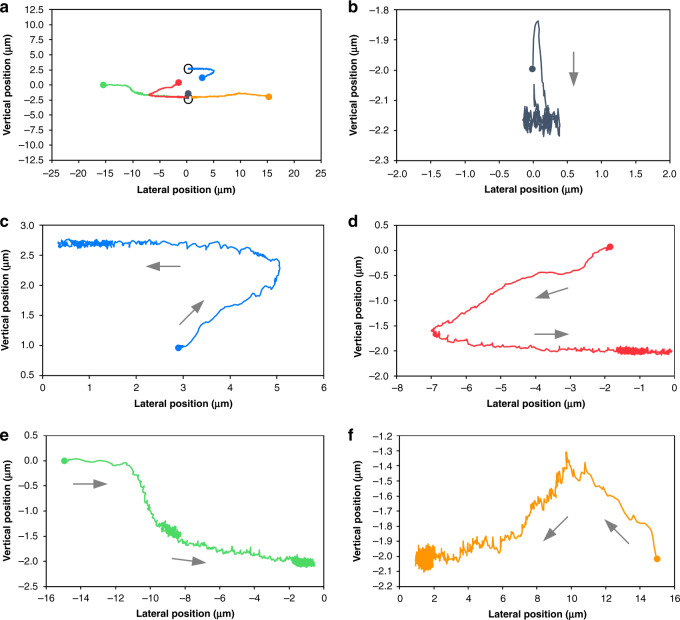
Smoothed particle hydrodynamics (SPH) simulations of the migration trajectories of the particles initially located in five representative positions within the channel cross section. The initial positions are marked by solid circles (mass centers of the 18.7-μm-diameter particles) with different colors. **a** An overview of the particle migration paths of the five particles within the channel cross section at *Re* = 50. The black open circles indicate the two final focusing positions. **b**–**f** Graphs showing the close-up details of the five migration paths. The arrows indicate the migration direction.

We applied computational modeling to the simulate transient motions of 3D particles and compared the predicted migration paths in the cross section with those from the experiments. Unlike the frequently used models for simulating force balance^28,34^, we employed the method of smooth particle hydrodynamics (SPH), which directly simulates the transient motion of particles and thus provides particle migration trajectories within the channel cross section. The motion of 18.7-μm-diameter particles initially located in five representative positions within the channel cross section was simulated at *Re* = 50. The particles were modeled one by one to avoid the influence of particle–particle interactions. The initial positions of the particles and the predicted trajectories are color-coded as shown in Fig. 7a, which is consistent with the findings in Fig. 6b.

As expected, all particles equilibrated in the two stable positions centered on the channel widths, which is in agreement with our experimental observations. The black particle initially near the channel center axis migrated rapidly toward the center of the bottom wall (Fig. 7b), similar to the particle in Fig. 3c. The blue particle initially off the center plane and horizontal centerline (Fig. 7c) underwent both horizontal and vertical displacements. A similar trajectory was observed for the red particle initially located in the vertical centerline (Fig. 7d), except that the horizontal migration toward the left sidewall was faster than the vertical migration toward the bottom wall (Movie S1). This result slightly deviates from the expected behavior (red path in Fig. 6a) as the particle displaces away from the vertical centerline. Nevertheless, this behavior is similar to other red paths in Fig. 6b. The green particle placed in the vertical centerline but near the left sidewall (Fig. 7e) showed the migration path more like the green one in Fig. 6a (Movie S2). Additionally, we simulated the orange particle in close proximity to the right sidewall but off the vertical centerline. This particle was found to migrate toward the vertical centerline as well as away from the right sidewall before taking on a path similar to the green one (Fig. 7f). Ultimately, all particles equilibrated in the two positions centered on the channel widths, which agrees with our experiments and the two-stage migration model.

## Discussion

### Force analysis of the migration pathways

The motion of a particle suspended in an inertial flow is governed by the net inertial force that induces cross-streamline migration^33^. Far away from the sidewalls, particle migration is dominated by the shear-induced lift force (*F*_s_), which drives the particles toward channel walls^33^. This is the Stage-I migration in our two-stage model^33^. However, the details of particle migration in this stage were not revealed previously due to the limitations of the PSV observation method that only yields the collective migration behavior of particles. Using high-speed imaging at the single-particle level in this work, we found distinct migration pathways that suggest a non-unidirectional shear-induced lift force depending on the particle location within the cross section.

To explain the heterogeneous migration pathways, we considered forces acting on the particles initially located at different positions in the channel cross section. Since it is not possible to experimentally decouple and measure all of the forces acting on the particles, our force analysis is based on the existing understanding of inertial microfluidics physics. Specifically, we consider three major forces that govern the migration dynamics in the inertial microfluidic channels: the shear-induced lift force, the wall-induced lift force, and the rotation-induced lift force^33^. The shear-induced lift force is due to fluid shear, and its direction is up the shear gradient (toward the channel wall)^44^. The wall-induced lift force becomes important only near the channel wall^45,46^. The rotation-induced lift force is an order of magnitude smaller than the shear-induced lift force, and its direction is down the shear gradient^33,47–49^. The rotation-induced lift force becomes important in close proximity to the channel wall where the shear rate is large^48^.

**Fig. 8:**
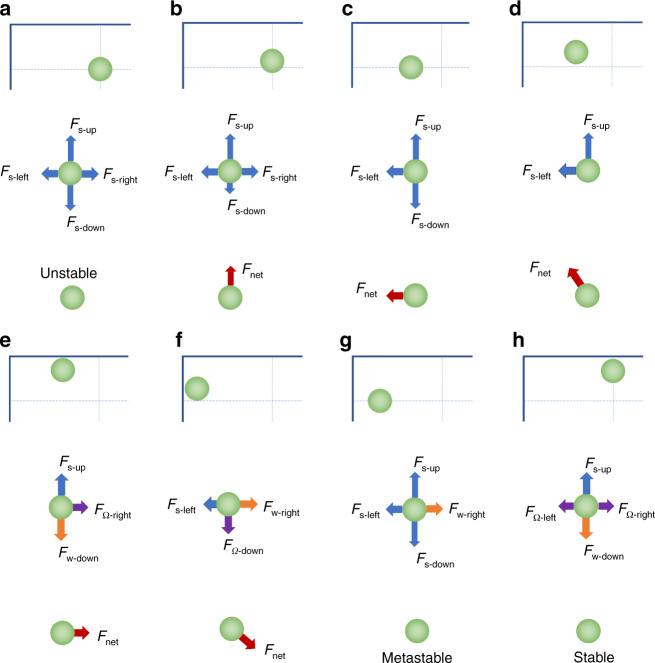
Mapping the inertial forces behind the heterogeneous migration behavior depends on the cross-sectional location. Shear-induced lift force (*F*_s_) dominates the particle migration when the particles are away from the channel walls and located in **a** the center axis, **b** the horizontal centerline toward the top wall, **c** the vertical centerline toward the left wall, and **d** the upleft quarter of the cross section. The wall-induced lift force (*F*_w_) and rotation-induced lift force (*F*_Ω_) come into play when the particles are in proximity to the channel walls: **e** near the top wall, **f** near the left sidewall, **g** slightly away from the left sidewall giving rise to a metastable position, and **h** horizontal centerline near the top wall giving a stable focusing position. Here, only a quarter of the rectangular cross section (first row) is present due to its symmetry. The second row shows all the inertial forces involved, and the net force is indicated in the third row

Using the symmetry of the rectangular cross section, we conducted a qualitative force analysis of four representative cases to explain the migration paths during Stage-I migration. In our analysis, we decompose the shear-induced lift force into its horizontal and vertical components in the rectangular cross section. Thus, depending on the cross-sectional location, the vertical component is directed either up or down; similarly, the horizontal component is directed either left or right. In the first case of a spherical particle initially located at the geometric center of the rectangular cross section (Fig. 8a), due to the physical size, the particle is subjected to the opposing components of the shear-induced lift force, which leads to a zero net force acting on the particle. As a result, the channel center axis can be a focusing position. However, this position is not stable because the force balance is too delicate. The instability stems from the divergent tendency of the shear-induced lift force, which scales with the square of the shear rate ($F_{\mathrm s}\sim \dot \gamma ^2$)^33^. The shear rate increases as the particle moves away from the center axis and is radially asymmetric. Thus, for a particle slightly displaced upward (Fig. 8b), the upward component of the shear-induced lift force (*F*_s-up_) increases, while the opposing component (*F*_s-down_) diminishes. The difference between these two components gives rise to the net lift force directed upward, driving the particle further away from the center. Similarly, for a particle slightly displaced to the left (Fig. 8c), *F*_s-left_ increases, and *F*_s-right_ decreases, causing the particle to continue its leftward migration. Such a tendency to move away from the center holds true for a particle slightly displaced both left and up (Fig. 8d), which explains the common migration pathways in the central region (Figs. 6 and 7c).

Practically, it is impossible to keep particles right in the center of the cross section as a disruption of the symmetry due to minute perturbations in the flow, tiny imperfections in the particle shape, and slight distortions of the channel cross section can displace the particle forever. Similarly, particles in the horizontal and vertical centerlines tend to move away due to the monotonous increase in the net shear-induced lift force once they are displaced. This finding explains the discrepancy between the predicted migration path (red path in Fig. 6a) and the path revealed in the simulation (Fig. 7d). The general tendency of particles moving to the long walls of the cross section stems from the radially asymmetrical distribution of the shear rate in the rectangular cross section where *w* > *h*. In the direction of the horizontal centerline (*y* = 0), the shear rate ($\dot \gamma = 2U_{\mathrm f}/h$) is larger than that ($\dot \gamma = 2U_{\mathrm f}/w$) along the vertical centerline (*z* = 0). Therefore, the shear force is generally larger in the vertical direction than in the horizontal direction ($F_{\mathrm s}\sim \dot \gamma ^2$).

When particles are in proximity to channel walls, Stage-II migration ensues as additional forces come into play. As a particle approaches the top channel wall, a wall-induced lift force (*F*_w_) arises normal to the wall, counteracting the upward shear-induced lift force (*F*_s-up_)^33^. As illustrated in Fig. 8e, the particle is subjected to the net force directed to the right. This net force is mainly due to the particle rotation and is termed the rotation-induced lift force (*F*_Ω_)^33^ in the two-stage model. There is likely a left component of the shear-induced lift force (*F*_s-left_) considering the radially distributed shear rate, but this component should be much smaller than the right component of the rotation-induced lift force (*F*_Ω-right_).

While these force interactions also apply to particles near the short walls, the migration dynamics are slightly different from the case of particles near the long walls. Here, the particle moves downward toward the vertical centerline while also moving away from the short wall (Fig. 8f). This is mainly due to the interaction of the wall-induced lift force and the shear-induced lift force. Since the shear rate ($\dot \gamma$) along the horizontal direction ($\dot \gamma = 2U_{\mathrm f}/w$) is smaller than in the vertical direction ($\dot \gamma = 2U_{\mathrm f}/h$), the shear-induced lift force (*F*_s-left_ in Fig. 8f) becomes relatively smaller in comparison with the shear lift force near the long sidewalls (*F*_s-up_). Since the wall-induced lift force is largely determined by the gap (*δ*) between the particle and the nearest wall (*F*_w_~*a*^3^/*δ*)^33^, it (*F*_w-right_) can exceed the weak shear-induced lift force (*F*_s-left_). The net of these two forces drives particles away from the wall, and the net force of all forces (Fig. 8f) explains the green migration path in Figs. 6a and 7e. Attributed to the convergent tendency of the rotation-induced lift force, the particle is driven to the vertical centerline, where it is further pushed away from the nearest wall by the wall-induced lift force (*F*_w-right_) until the diminishing *F*_w-right_ is balanced by *F*_s-left_ as a result of the increased *δ*.

This dynamic change in the forces leads to the potential focusing position shown in Fig. 8g. However, this position is metastable as the shear-induced lift force (here, either *F*_s-up_ or *F*_s-down_) tends to drive the particle away from the vertical centerline toward the long walls (divergent tendency), should there be a slight vertical displacement caused by perturbations in the flow or defects in the channel symmetry. As a result, particles near the short walls eventually migrate to the long walls, where they are driven to the final equilibrium positions in the horizontal centerline near the long walls (Fig. 8h). The two positions in the horizontal centerline near the long walls are stable because a change in one force induces a similar change in the opposing force. For example, if the shear-induced lift force increases (*F*_s-up_ ↑), it pushes particles closer to the wall (*δ* ↓), and then the wall-induced lift force also increases (*F*_w-down_ ↑) to ensure the zero net force in the vertical direction; if the particle is displaced to the left horizontally, the rotation-induced lift force (*F*_Ω-right_) increases to push the particle back to the equilibrium position.

## Concluding remarks

Our revelation of the migration paths and the underlying force interactions has a broad implication with regard to the dynamics of inertial migration. First, the metastable positions suggested by the opposing migration paths (red and green in Fig. 6a) explain the temporary presence of the two-side streams observed in addition to the stable stream in the horizontal centerline of rectangular microchannels^12,33^. Undergoing these longer migration paths, particles near the two short sidewalls aggregate in the two metastable positions (Fig. 8g) before reaching the final equilibrium positions near the centers of the long walls (Fig. 8h), giving rise to the side streams observed in top-view imaging. Confocal images showing that particles aggregated near sidewalls exhibited an oval shape extending from the sidewall toward the channel center axis in the vertical centerline^20^, also corroborating our assumptions of the existence of metastable positions.

Second, the two metastable positions near channel sidewalls can become stable if the force balance changes. The lateral positions of these metastable positions are dependent on the interaction of *F*_w-right_ and *F*_s-left,_ and the instability of these metastable positions stems from the divergent tendency of the vertical shear-induced lift forces (*F*_s-up_ and *F*_s-down_). Increasing *F*_s-left_ by increasing the flow rate (and thus higher *Re*) elevates the shear rate ($F_{\mathrm{s}}\sim \dot \gamma ^2$) and drives the metastable positions closer to the sidewalls where *F*_Ω-down_ and *F*_Ω-up_ become dominant in the vertical direction. Due to the convergent tendency of *F*_Ω_, the metastable positions become stable as the forces are balanced in the way of the two equilibrium positions near the centers of the long walls (Fig. 8h). Indeed, these new stable positions were reported by Liu et al.^34^ for 15-μm-diameter particles in a 100 μm × 50 μm channel when the flow rate increased from *Re* = 100 to *Re* = 200. Similarly, we observed the side streams for 10-μm-diameter particles at *Re* = 100 but not at *Re* = 50 in our current channel (Fig. S2).

Alternatively, the metastable positions can become stable when particle size is reduced, since both *F*_w_ and *F*_s_ are strongly dependent on particle size (*F*_w_ ~ *a*^3^/δ and *F*_s_ ~ *a*^2^)^33^. A smaller particle diameter leads to a greater reduction in *F*_w-right_ than in *F*_s-left_ if the gap δ remains unchanged. The net force then drives the smaller particle toward the sidewalls, reducing the gap *δ*, which increases *F*_w-right_ to balance *F*_s-left_ again. A smaller *δ* means that particles are closer to the sidewalls and thus become confined vertically by *F*_Ω_, stabilizing the metastable positions. Such a size-dependent stability of the metastable positions was reported for the 100 μm × 50 μm channel, where 5-μm-diameter particles occupied two-side focusing positions (in the vertical centerline) in addition to the other two positions in the horizontal centerline, while 15-μm-diameter particles were only observed in the latter two positions^34^. Similarly, we observed side streams for 7.32-μm-diameter particles but not for 10-μm-diameter particles at *Re* = 50 in our current channel. This size-dependent stability of the metastable positions also explains the complete separation of 10- and 20-μm-diameter particles in a straight channel that we demonstrated by changing the channel aspect ratio^29^.

Finally, the analysis of the migration paths and forces involved explain and predict the inertial migration dynamics in other cross-sectional geometries. For example, in a square channel, the force balance is identical for the positions near the center of each wall, giving rise to four stable focusing positions. On the other hand, particles located in the two diagonals will be driven to the four corners due to the identical magnitude of the vertical and horizontal components of the shear-induced lift force as a result of the symmetrical distribution of the shear rate. At a moderate *Re*, particles will be pushed back to the center positions once they are close to the corner as a result of the rotation-induced lift force. However, if the shear-induced lift force increases excessively, as in the case of large *Re* (e.g., *Re* = 300)^50^, then it can force particles even closer to the corners where it can be balanced by the combination of the wall-induced lift and the rotation-induced lift forces, both acting along channel walls and away from the corner. In this case, the four corner positions can become stable in addition to the four positions near the centers of the four walls. Similarly, depending on the interactions of the three forces that can be modulated by the channel geometry, which changes the distribution of the shear rate, particles can be focused into three positions or six positions in a triangular channel^30,31^.

## Supplementary information

Supplemental Material

Movie S1

Movie S2
