# Supplementary material for: Mapping inertial migration in the cross section of a microfluidic channel with high-speed imaging
Source: Microsyst Nanoeng. 2020 Nov 16;6:105. doi: 10.1038/s41378-020-00217-y (PMC8433405; doi:10.1038/s41378-020-00217-y)
Supplement: Supplementary file 1 — Supplemental Material [file 41378_2020_217_MOESM1_ESM.pdf]

Supplemental Information

Mapping inertial migration in the cross-section of a microfluidic channel with high speed imaging

Jian Zhou, Zhangli Peng and Ian Papautsky
Department of Bioengineering, University of Illinois at Chicago, USA

This supplemental information includes (1) supplemental figures (Fig. S1, Fig. S2 and Fig. S3) and related discussion, and (2) movies (Movie S1 and Movie S2) showing 3D particle migration inside the microchannel. The movies were obtained from simulation using smoothed particle hydrodynamics (SPH). The red markers in the movies are used to indicate particle spinning. We used slightly modified versions of the USER-SPH package and the RIGID package in Large-scale Atomic/Molecular Massively Parallel Simulator (LAMMPS), which are publicly available on LAMMPS website (https://lammps.sandia.gov/).

Description and discussion of Fig. S1

Stacked images for the first four millimeters of channel downstream length confirm the calculation of focusing length, which is less than 3 mm (Fig. S1). While most investigators reporting on inertial migration use fluorescent particle streak velocimetry, herein we use bright-field high-speed imaging to capture individual particles within the 1 mm field of view. This is because fluorescent imaging may conceal details of rare events, such as the temporary or weak side streams we observed in our previous work[12,33]. Both side-view and top-view images confirm that tight focusing of 18.7-µm particles was achieved in less than 3 mm downstream length. While non-uniform distribution of particle size was observed, all particles were in proximity to sidewalls (long faces of channel cross-section) in side-view and entrained in the

channel center in top-view at 3 mm downstream position, which is in agreement with our two-stage migration model[33].

Nevertheless, some inconsistencies were also noted in our side-view images. While the majority of particles already reached sidewall positions after the first 1 mm downstream length, a few particles (indicated by red arrows in Fig. S1) remained in the middle of the channel in side-view, which contradicts our expectations that particles should complete their first-stage migration toward sidewalls in a few hundred microns of channel length. In fact, inertial migration in the existing literature is legitimately corroborated either by recording lateral positions of a number of particles at different downstream locations (PSV)[33,51] or by quantifying outcomes of bifurcation outlets[9]. Trajectories of individual particles within a microchannel are the direct evidence that is missing in the current literature. Such evidence will provide a wealth of information regarding the migration dynamics of inertial focusing, which may help reconcile the prior inconsonance and ensue new findings that further our understanding of migration mechanism. We used high-speed imaging to obtain the missing direct evidence and to ascertain inertial migration at single-particle level.

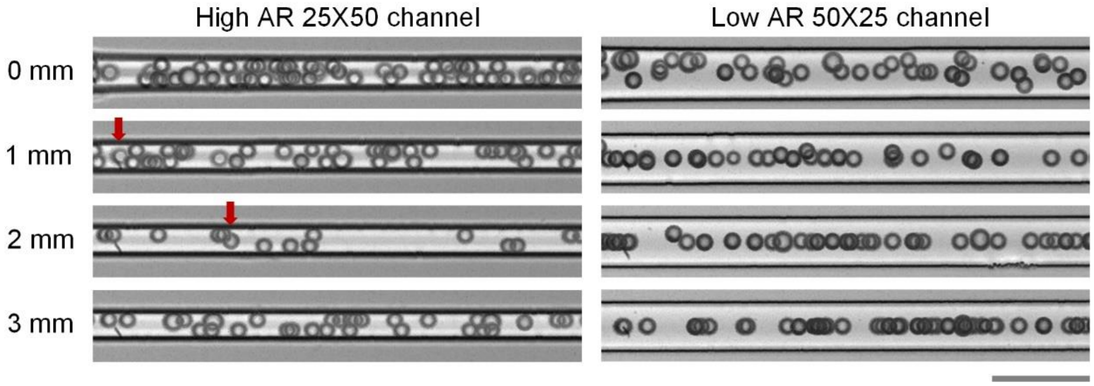

Fig. S1. Focusing of 18.7 μm diameter particles in rectangular microchannels at *Re* = 50 at successive downstream positions. The majority of particles focus in two equilibrium positions in the high AR channel (left) and along centerline in the low AR channel (right). Each image is a stack of >1,000 frames to illustrate the collective migration behavior of particles. Downstream position was measured from the left edge of each image. Red arrows indicate particles that remained in the channel center. Frame rate was 125 fps. Scale bar is 100 μm.

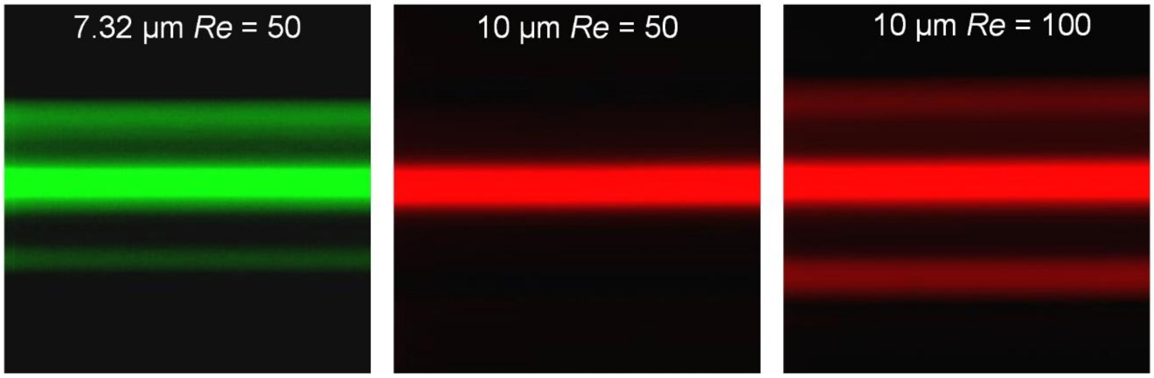

Fig. S2. Presence of two side streams depends on particle size and Reynolds number in the 50 µm × 25 µm channel with length of 10 mm. The side streams were observed for the 7.32 µm diameter particles, but were not present for the 10 µm diameter particles at *Re* = 50. The two side streams re-emerged for the 10 µm diameter particles at *Re* = 100.

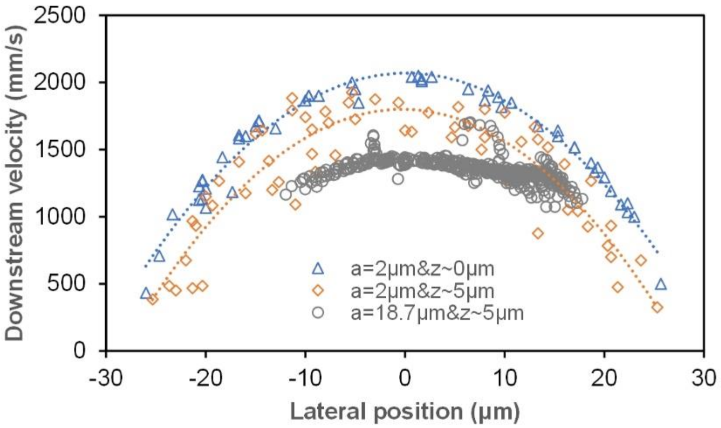

Fig. S3. Distribution of fluid and particle velocities. Fluid velocities near the vertical centerline and about 5 µm away from the vertical centerline were measured using 2 µm-diameter particles as tracers. Grey open circles are the velocities of 18.7 µm-diameter particles near the top or bottom walls, which is about 5~6 µm away from the vertical centerline.
